# SGI-4 in Monophasic *Salmonella* Typhimurium ST34 Is a Novel ICE That Enhances Resistance to Copper

**DOI:** 10.3389/fmicb.2019.01118

**Published:** 2019-05-24

**Authors:** Priscilla Branchu, Oliver J. Charity, Matt Bawn, Gaetan Thilliez, Timothy J. Dallman, Liljana Petrovska, Robert A. Kingsley

**Affiliations:** ^1^Quadram Institute Bioscience, Norwich, United Kingdom; ^2^Gastrointestinal Bacteria Reference Unit, National Infection Service, Public Health England, London, United Kingdom; ^3^Animal and Plant Health Agency (APHA), Addlestone, United Kingdom; ^4^School of Biological Sciences, University of East Anglia, Norwich, United Kingdom

**Keywords:** *Salmonella*, monophasic, integrative conjugative element, SGI-4, copper resistance

## Abstract

A multi drug resistant *Salmonella enterica* 4,[5],12:i- of sequence type 34 (monophasic *S*. Typhimurium ST34) is a current pandemic clone associated with livestock, particularly pigs, and numerous outbreaks in the human population. A large genomic island, termed SGI-4, is present in the monophasic Typhimurium ST34 clade and absent from other *S*. Typhimurium strains. SGI-4 consists of 87 open reading frames including *sil* and *pco* genes previously implicated in resistance to copper (Cu) and silver, and multiple genes predicted to be involved in mobilization and transfer by conjugation. SGI-4 was excised from the chromosome, circularized, and transferred to recipient strains of *S*. Typhimurium at a frequency influenced by stress induced by mitomycin C, and oxygen tension. The presence of SGI-4 was associated with increased resistance to Cu, particularly but not exclusively under anaerobic conditions. The presence of *silCBA* genes, predicted to encode an RND family efflux pump that transports Cu from the periplasm to the external milieu, was sufficient to impart the observed enhanced resistance to Cu, above that commonly associated with *S*. Typhimurium isolates. The presence of these genes resulted in the absence of Cu-dependent induction of *pco* genes encoding multiple proteins linked to Cu resistance, also present on SGI-4, suggesting that the system effectively limits the Cu availability in the periplasm, but did not affect SodCI-dependent macrophage survival.

## Introduction

*Salmonella enterica* serovar Typhimurium (*S.* Typhimurium), including monophasic variants, accounts for approximately 25% of all human cases of non-typhoidal *Salmonella* (NTS) infection in Europe, and is widespread in multiple animal reservoirs (Hugas and Beloeil, 2014; Animal and Plant Health Agency [APHA], 2017; Branchu et al., 2018). The epidemiological record of human multidrug-resistant (MDR) *S.* Typhimurium infections in Europe is characterized by successive waves of dominant MDR variants that persist for 10–15 years (Rabsch et al., 2001; Rabsch, 2007). *S.* Typhimurium DT104 emerged around 1990, becoming a globally pandemic clone that affected numerous domesticated and wild animal species (Threlfall, 2000). Subsequently, in 2007, a monophasic *S*. Typhimurium variant (*S*. 4,[5],12:i-) of sequence type 34 (ST34) emerged in European pig populations and spread globally (hereafter referred to as monophasic *S*. Typhimurium ST34; Hauser et al., 2010; Antunes et al., 2011; Hopkins et al., 2012; Arguello et al., 2013; Mourao et al., 2015; Andres-Barranco et al., 2016; Bonardi, 2017). The mechanisms that drive succession of *S*. Typhimurium variants are not known, but selection by commonly used antibiotics is unlikely since successive variants share similar AMR profiles (Rabsch, 2007): ACSSuT (ampicillin, chloramphenicol, streptomycin, sulphonamide, tetracycline) for *S*. Typhimurium DT104 and ASSuT for monophasic *S*. Typhimurium ST34.

Cu is both an essential nutrient, due to its role as a cofactor in multiple enzymes in all aerobic organisms, and highly toxic due to its ability to displace iron from iron-sulfur clusters in dehydratases (Macomber and Imlay, 2009). To reduce toxicity, bacteria control the amount of free Cu in the cytoplasm and periplasm using transport systems, and by the oxidation of cuprous (Cu^1+^) to less toxic cupric (Cu^2+^) ions (Rensing and Grass, 2003). *Escherichia coli*, a close relative of *Salmonella*, encodes multiple transport systems on its chromosome to maintain Cu homeostasis. To overcome the toxicity of Cu, *E. coli* transports Cu from the cytoplasm into the periplasm via a P_1B_-type ATPase, CopA, and from the cytoplasm and periplasm to the external milieu via a multicomponent Cu RND family efflux pump, CusCFBA, and oxidizes cuprous ions by the action of the multi-Cu oxidase, CueO (Grass and Rensing, 2001; Franke et al., 2003). CueO and CopA, which are co-regulated by the cytosolic CueR, are the primary Cu homeostasis systems active during aerobic growth (Stoyanov et al., 2001), while CusCFBA is important during anaerobic growth, under the transcriptional control of the periplasmic CusRS two component regulator (Outten et al., 2001). In addition, some *E. coli* isolated from Cu-rich environments have additional plasmid-encoded genes *pcoABCDRSE*, that encode several proteins including a multicopper oxidase system which is active in the periplasm, Cu transporters, and a Cu binding protein. The *pco* locus is regulated by a two-component system (PcoR/PcoS) with a sensor kinase that extends into the periplasm (Brown et al., 1995; Rensing and Grass, 2003).

Cu homeostasis in the genus *Salmonella* appears to be fundamentally different from that in *E. coli* due to the deletion of the *cusRSCFBA* genes from the chromosome noted in a number of commonly used lab strains (McClelland et al., 2001; Espariz et al., 2007; Pontel and Soncini, 2009; Fookes et al., 2011). Therefore, although *Salmonella* encode CopA and CueO, the lack of the CusCFBA RND family efflux pump means they lack the ability to transport Cu out of the cell entirely, which is likely to have a significant impact on the distribution of Cu within the cell. However, plasmid-borne *silRSECBAP* genes have been described in a *S*. Typhimurium strain associated with an outbreak in burn patients that had been treated topically with silver nitrate; these genes encode an RND family efflux pump that is closely related to the CusCFBA system and conferred resistance to silver and Cu (McHugh et al., 1975; Gupta et al., 1999). Although the *sil* and *cus* genes are generally absent from the whole genome sequence of reference strains of *Salmonella*, these genes have been reported in multiple distinct monophasic *S*. Typhimurium and *S*. Rissen clones associated primarily with pigs in the past two decades (Mourao et al., 2015; Campos et al., 2016; Mastrorilli et al., 2018).

When the whole genome sequence of monophasic *S*. Typhimurium ST34 was compared with other *S*. Typhimurium whole genome sequence, an 80kb genomic island was identified and designated as *Salmonella* genomic island 4 [SGI-4, note addendum for nomenclature change from SGI-3 (Petrovska et al., 2016)]; this genomic island was present in over 95% of monophasic *S*. Typhimurium ST34 strains from the United Kingdom and Italy, but absent from a diverse collection of *S*. Typhimurium including DT104 (Petrovska et al., 2016). Ancestral reconstruction analysis was consistent with acquisition of SGI-4 by horizontal transfer concomitant with clonal expansion of monophasic *S*. Typhimurium ST34, and rare sporadic loss of the genetic island (Petrovska et al., 2016). SGI-4 contained three clusters of genes predicted to be involved in resistance to Cu and silver or arsenic metal ions (Petrovska et al., 2016).

Here we addressed the hypothesis that SGI-4 is a mobile genetic element (MGE) that encodes multiple metal ion resistance determinants that alter the growth of *Salmonella* in concentrations of Cu relevant to the host and farm environments. Furthermore, we test the hypothesis that transferable Cu and silver resistance genes alter expression of endogenous Cu homeostasis genes that sense Cu levels in the periplasm and that this affects SocCI-mediated survival in macrophages.

## Materials and Methods

### Bacterial Strains and Growth Conditions

*Salmonella* Typhimurium strain SL1344 was isolated from a calf in 1973 as described previously (Kroger et al., 2012). Monophasic *S*. Typhimurium ST34 strains (S04698-09, S04332-09, L00857-09, S05092-07, S06578-07, and L0938-09), *S*. Typhimurium U288 strains S01960-05, S05968-02 and 11020-1996, *S*. Typhimurium DT104 strains (NCTC13348, 4582-1995 and S00914-05), and two strains that were closely related to strain SL1344 (9115-1996 and 6164-1997) were isolated from various host species and their whole genome sequences were determined as described previously (Mather et al., 2016; Petrovska et al., 2016). For planktonic culture, bacteria were inoculated from single colonies subcultured on LB agar plates into 10ml of growth medium and incubated at 37^o^C in aerobic (normal atmospheric) conditions, or microaerobic (10% CO_2_, 5% H_2_, 5% O_2_, and 80% N_2_) or anaerobic (10% CO_2_, 10% H_2_, and 80% N_2_) conditions using a Whitely A95 Anaerobic Workstation (don whitley scientific). Strains of *S*. Typhimurium or monophasic *S*. Typhimurium in which specific genes were replaced with either the *cat* gene conferring chloramphenicol resistance or the *aph* gene conferring resistance to kanamycin were constructed using allelic exchange methodology as described previously (Datsenko and Wanner, 2000). Briefly, pairs of oligonucleotide primers specific for amplification of the *cat* gene or the *aph* gene from plasmids pKD3 or pKD4 were synthesized with 50 nucleotide sequences on the 3′ end that were identical to sequence immediately proximal to the ATG start and distal to the stop codon of each gene targeted for deletion (Supplementary Table S1). Bacterial strains were routinely cultured in Luria Bertani broth (Oxoid) supplemented with 0.03 mg/l chloramphenicol or 0.05 mg/l kanamycin as appropriate.

### Sequence Analysis

A gene model for SGI-4 was constructed using Prokka (Seemann, 2014) with a minimum open reading frame (ORF) size of 100. Annotation of SGI-4 was achieved by aligning ORF sequences to those in the NCBI database using BLASTn to identify genes with the greatest sequence identity.

Phylogenetic trees from whole genome sequence data were constructed and single nucleotide polymorphisms (SNPs) identified in the whole genome sequences by aligning reads using BWA-MEM (Li, 2013), variant calling with Freebayes (Garrison and Marth, 2012) and SNP filtering using vcflib/vcftools (Danecek et al., 2011), combined as a pipeline using Snippy v3.0.^1^ Maximum-likelihood trees were constructed using a general time-reversible substitution model with gamma correction for amongst-site rate variation with RAxML v8.0.20 with 1000 bootstraps (Stamatakis, 2006). Representative isolates of *S*. Typhimurium phage types described previously (Petrovska et al., 2016), and whole genome sequences of *S*. Typhimurium isolates from routine clinical surveillance by Public Health England are available in public databases with accession numbers reported in Supplementary Table S2.

Candidate SGI-4-like elements (SLEs) were identified in genome assemblies in the NCBI non-redundant sequence database (accessed June 2018) by aligning to SGI-4 excluding the *ars*, *sil*, and *pco* loci, using discontiguous megaBLAST. This analysis identified nucleotide sequences from *Edwardsiella ictaluri* (accession CP001600, 326500..380800) *Erwinia tracheiphila* (accession CP013970, 1856073..1916737), *Enterobacter cloacae* (accession CP012162, 4040884..4152906), *Enterobacter hormaechei* (accession CP010376, 3932000..4028800), *Enterobacter hormaechei* (accession CP012165, 395200..536000), *Pluralibacter gergoviae* (accession CP009450, 1604785..1778603) and *Salmonella* Cubana (accession CP006055, 4214040..4311200). For reconstruction of the phylogeny of putative SLEs, sequence were aligned using clustalW-2.1 (Larkin et al., 2007) to determine sequence identity and conserved regions. A maximum likelihood tree was constructed from aligned nucleotide sequence using RAxML (Stamatakis, 2006). The relationship of SLEs was also investigated by determining the proportion of each SLE aligned by carrying out a pair-wise comparison with discontinuous megaBLAST (Morgulis et al., 2008). Each SLE was used as the query sequence against each SLE as the subject to determine the percent of sequence that aligned and the mean percent nucleotide sequence identity.

For reconstruction of a maximum likelihood tree to investigate the relationship of the SGI-4 encoded and previously described *silRSECBAF* and *cusRSCFBA* genes, sequence extracted from 17 whole genome sequences was aligned using ClustalW-2.1 (Larkin et al., 2007) and a tree constructed using 1000 bootstraps and the GTRCAT substitution matrix with RAxML-8.0.22 software (Stamatakis, 2006). The loci were from 15 Cu homeostasis and silver resistance island (CHASRI) sequences (Staehlin et al., 2016), of which nine were chromosomal and six on plasmids, as well as the originally described *silRSECFBAP* from *S*. Typhimurium plasmid pMG101, and chromosomal *cusSRCFBA* from *E. coli* K-12.

To estimate the distribution of the *silA* gene in 926 *Salmonella* whole genome sequences, one from each eBurst group (similar to serovar groups) and therefore representing the diversity of the genus (Alikhan et al., 2018), query sequence was aligned using BLASTn with an 80% coverage threshold and 2,000 maximum hits. BIGSI (Bradley et al., 2019) was used to query the presence of the *silA* and *invA* genes in over 450,000 bacterial sequence entries in the European Nucelotide Archive (ENA; accessed on March 29, 2019 with an api url search).

### Determination of SGI-4 Transfer *in vitro*

In order to provide a convenient selectable marker for the presence of SGI-4, we constructed a strain of monophasic *S*. Typhimurium S04698-09 in which the *bar* gene on SGI-4 was replaced by the *cat* gene (S04698-09 SGI-4 Δ*bar*::*cat*), conferring resistance to chloramphenicol (Supplementary Table S1). To provide a selectable marker for the recipient strain, we constructed a strain of *S*. Typhimurium SL1344 in which the *copA* gene was deleted and replaced by an *aph* gene, conferring resistance to kanamycin (Supplementary Table S1). Donors and recipients were cultured in LB broth for 18 h at 37°C with shaking. The OD_600nm_ of each culture was adjusted to 0.1 with fresh LB broth and 2.5 ml of each added to a 50ml tube and incubated for 18 h at 37°C with shaking in aerobic or anaerobic atmosphere, and in the presence or absence of 0.5 mg/l mitomycin C. The number of CFUs per ml of donors and recipients were quantified by culturing serial dilutions on LB agar supplemented with 0.03 mg/l chloramphenicol or 0.05 mg/l kanamycin, respectively. The presence of SGI-4 Δ*bar*::*cat* in recipient strains was quantified by serial dilution on LB agar supplemented with chloramphenicol and kanamycin. Transfer frequency was defined as the number of recipients containing SGI-4 Δ*bar*::*cat* as a proportion of donor cells in the culture. To determine whether transconjugant recipient strains contained SGI-4 in the same chromosomal location as the donor, the predicted right junction was amplified using primers that annealed on either side of the right junction of SGI-4 by PCR (Supplementary Table S1). To detect circularization of SGI-4 after excision, outward facing primers that annealed at the left and right junction of SGI-4 were used for PCR amplification (Supplementary Table S1). The sequence of the amplicons was determined by Sanger sequencing using the same primers (Eurofins sequencing service) and aligned to the genome of strain S04698-09.

### Determination of Minimal Inhibitory Concentration (MIC) for Cu Sulfate

Fifteen bacterial strains (monophasic *S*. Typhimurium ST34 strains S04698-09, S04332-09, L00857-09, S05092-07, S06578-07, L0938-09, and *S*. Typhimurium strains S01960-05, S05968-02, 11020-1996, NCTC13348, 4582-1995, S00914-05, SL1344, 9115-1996, and 6164-1997) were cultured for 18 h in LB broth at 37°C with shaking. A stock solution of 20 mM CuSO_4_ in LB broth and 25 mM HEPES pH7 using NaOH. Serial dilutions in LB broth (pH7) were performed to generate a range of concentrations in 1mM intervals from 1 to 20 mM CuSO_4_. 0.2 ml of LB broth buffered with 25mM HEPES pH7 and containing a range of concentrations of Cu sulfate were added to a polystyrene 96-well plate (Nunc) and incubated at 37°C for 24 h in normal atmospheric, microaerobic, or anaerobic conditions to equilibrate. After 24 h, each well was inoculated with 1 × 10^7^ colony-forming units (CFUs) of the test bacterial strain cultured for 18 h in 10 ml LB Broth with shaking. Plates were incubated at 37°C for 24 h in normal atmospheric, microaerobic, or anaerobic conditions. The OD_600nm_ of each well was measured using a BIORAD Benchmark Plus microplate spectrophotometer. The MIC was defined as the mean concentration of Cu sulfate for which the OD_600nm_ of the culture was <0.2 from four biological replicates.

### Quantitative Real Time PCR Expression Analysis

Expression relative to transcript abundance of a constitutively expressed housekeeping gene was determined as previously described (Branchu et al., 2014). Total RNA was extracted from 2 ml samples of overnight cultures of S04698-09 and S04698-09Δ*silCBA* strains grown in LB broth supplemented with 20, 100, 200 μM CuSO_4_, or without CuSO_4_ supplementation to mid-log phase (OD_600nm_ of 0.2) in anaerobic atmosphere (*pcoA*) or aerobic atmosphere (*copA*). Bacteria were harvested by centrifugation and RNA extracted using a FastRNA^TM^ spin kit for microbes (MPBio) according to the manufacturer’s instructions. RNA was treated with a TURBO DNA-free^TM^ kit (ambion, life technologies^TM^) according to the manufacturer’s instructions before being reverse-transcribed using the QuantitTect^®^ Reverse Transcription kit (Qiagen^®^). The resulting cDNA or serial dilutions of a known quantity of genomic DNA for generation of standard curves were amplified using a QuantiFast^®^ SYBR^®^ Green PCR kit (Qiagen^®^) with specific primers (Supplementary Table S1) for *copA* or *pcoA* test genes, and *rpoD* as a control housekeeping gene using the Applied Biosystems^®^ 7500 real-time PCR system. The expression of *copA* or *pcoA* is presented relative to transcript abundance of the *rpoD* gene.

### RAW264.7 Macrophage Survival Assay

Murine macrophages (RAW 264.7, ATCC, Rockville, MD) were grown in minimum essential medium (Sigma Aldrich) supplemented with 10% fetal bovine serum, L-glutamine (2 mM), and 1 × nonessential amino acids. For infection studies, 2 × 10^5^ RAW 264.7 cells were seeded per well into 24-well plates and incubated at 37°C for 48 h. Cells were infected with S04698-09 wild-type strain, S04698-09Δ*sodCI*, S04698-09Δ*silCBApco* or S04698-09Δ*sodCI*Δ*silCBApco* from an overnight culture in LB broth at a multiplicity of infection of 20. Plates were centrifuged at 1500 rpm for 3 min followed by incubation of the cells for 30 min. The medium was then exchanged for a fresh one of the same compositions with the exception of the addition of gentamicin (100 μg/ml) to kill the extracellular bacteria. After either 2 or 24 h, the cells were washed with PBS and lysed with 1% Triton in PBS. The number of bacterial CFUs was determined by culturing lysate serial dilutions on LB agar. The data were expressed as the proportion of CFUs at 24 h relative to the CFU determined at 2 h.

### Statistical Analysis

Where indicated in each figure with lines and asterisks, the Mann-Whitney *U* test was used to test the null hypothesis that randomly selected values in a sample were equally likely to be greater or smaller than from a second sample, using an alpha level of 0.05 to reject the null hypothesis.

## Results

### SGI-4 Is a Member of a Novel Family of Integrative Conjugative Elements (ICE)

**FIGURE 1:**
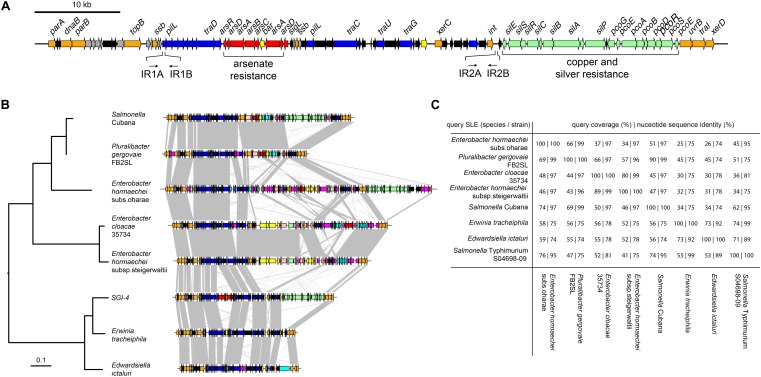
Genetic map of *Salmonella* Genomic Island 4 (SGI-4) and phylogenetic relationship and gene synteny with SLEs. **(A)** Genetic diagram of *Salmonella* Genomic Island 4 (SGI-4); filled arrows indicate open reading frames with predicted functions based on sequence similarity for integration, excision and DNA processing (orange), type 4 secretion systems and conjugal transfer (blue), Cu and silver resistance (green), arsenic resistance (red), genes of unknown function commonly associated with ICE (gray), genes of unknown function not commonly associated with ICE (black). **(B)** Mid-point rooted maximum likelihood tree of eight SGI-4-like elements (SLEs) constructed using sequence variation in the core nucleotide sequence alignment of SLEs from diverse Enterobacteriaceae and genetic diagram of SLEs with regions of >75% nucleotide sequence identity (gray shading); filled arrows indicate open reading frames with the same designations as **(A)**, with additional functions, cadmium, cobalt, zinc or mercury resistance (pink), transposase and insertion elements (purple), and a repressor protein lexA (brown). **(C)** Pair-wise sequence comparison of SLEs indicating the percentage of sequence exhibiting >60% identity and the mean nucleotide sequence identity of the shared sequence.

We investigated the presence of candidate genes within the SGI-4 coding for proteins capable of enabling mobilization of SGI-4 to recipient bacteria. The DNA excision, DNA processing, and conjugative transfer mechanisms we considered are common to MGEs such as ICE, also known as conjugative self-transmissible integrative (CONSTIN) elements (Hochhut and Waldor, 1999), integrative mobilizable elements (IMEs), and *cis*-mobilizable elements (CIMEs; Wozniak and Waldor, 2010). A number of ORFs that exhibited sequence similarity to DNA processing enzymes were identified on SGI-4 by sequence alignment to available databases (Figure 1A and Supplementary Table S3). Sequences of two of these exhibited similarity to site-specific tyrosine recombinases *xerC* and *xerD*, which are predicted to mediate integration and excision. However, we were unable to identify a recombination direction factor (RDF), which are known to exhibit little sequence conservation. ORF86 had sequence similarity to *traI* that encodes a relaxase protein. This relaxase protein is capable of binding to dsDNA at the origin of transfer (oriT) and inducing a single strand nick in conjunction with putative *uvrB* DNA helicase (ORF85) and *topB* topoisomerase (ORF 13), this facilitates unwinding of the DNA helix (Salyers et al., 1995). At the left end of SGI-4, *parA* and *parB* orthologs (ORFs 1 and 5) encode putative chromosome partitioning proteins. SGI-4 also contained a number of ORFs with similarity to genes encoding components of type IV secretion systems (T4SSs) involved in conjugative transfer of DNA: ORF26 of SGI-4 encoded a putative type IV coupling protein (T4CP), TraD, which initiates conjugal transfer; ORF51 encodes a putative inner membrane protein, TraG, for T4SS stabilization; ORF53 encodes a TraU ortholog involved in pilus assembly; ORF19 encodes a PilL F-type pilus protein; and ORF45 encoding an F-pilus assembly protein. These ORFS were present within several apparent operons (ORFs 14–27, 37–46, 48–53), consisting of multiple genes encoding proteins with no significant similarity to proteins in available databases but often associated with MGEs (Figure 1A and Supplementary Table S3). We were unable to identify a putative *oriT* sequence in SGI-4 using oriTfinder (Li et al., 2018). However, two pairs of inverted repeats, each with 80% identity to one another, were present in ORF19 and in an intergenic region between ORF64 and ORF65.

To determine whether SGI-4 was similar to known MGEs we aligned the sequence of SGI-4 with non-redundant nucleotide sequences in the NCBI database using discontinuous megaBLAST. Seven assembled contiguous sequences in the NCBI database contained a core set of ICE genes with a modular arrangement with complete synteny to SGI-4, none of which had been described previously as MGEs (Figure 1B). These exhibited at least 75% nucleotide sequence identity with over 40% of SGI-4, and we therefore refer to these as SGI-4-like elements (SLEs). SLEs were inserted in the genome adjacent to a phenylalanine phe-tRNA in strains from a diverse range of Enterobacteriaceae, indicating this was the common attachment site (attB/attP). SGI-4 was most closely related to SLEs from *Erwinia tracheiphila* and *Edwardsiella ictaluri*, and more distantly related to SLEs from two *Enterobacter hormachei* strains from subspecies *oharae* and *steigerwaltii*, *Enterobacter cloacae, Pluralibacter gergoviae* and an SLE present in a strain of *Salmonella enterica* serovar Cubana (Figure 1B,C). All shared a number of regions of at least 75% sequence identity and synteny that encoded putative DNA processing enzymes or components of a T4SS, and were interspersed amongst apparent cargo genes involved in diverse functions capable of modifying the phenotype of the host bacterium.

**FIGURE 2:**
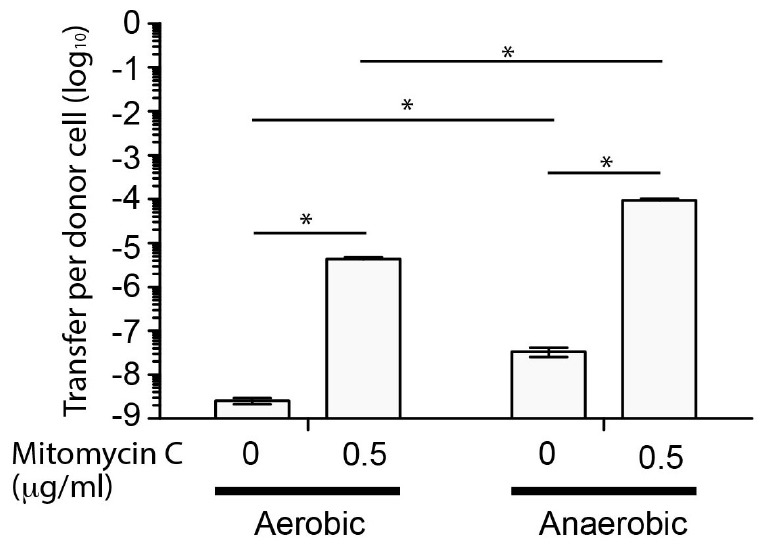
Transfer frequency of SGI-4::*cat* to *S*. Typhimurium during co-culture is enhanced by mitomycin C and anaerobic conditions. Bars indicate the mean number of chloramphenicol resistant CFU of *S*. Typhimurium strain SL1344 per CFU of monophasic *S*. Typhimurium ST34 strain S04698-09 SGI-4 *silCBA*::*cat*, following co-culture. The mean from six biological replicates ± standard deviation are shown. ^∗^Indictaes that groups indicated by lines were significantly different (*p* < 0.05).

Since SGI-4 encoded many of the genes normally associated with ICE, we determined whether it was capable of mobilization to a recipient *S*. Typhimurium (strain SL1344) during co-culture. To enable identification of recipients containing SGI-4 we inserted a chloramphenicol resistance gene (*cat*) in the arsenic resistance locus of SGI-4. Transfer frequency was low under aerobic culture conditions (<1 × 10^-8^ CFU per donor CFU) but was substantially increased in the presence of mitomycin C, as described previously for the SXT ICE (Beaber et al., 2004), or by culture in anaerobic conditions (Figure 2). Mitomycin C and anaerobiosis had an additive effect on transfer since the transfer rate in anaerobic conditions with mitomycin C was significantly higher (*p* < 0.005) than any other condition tested (∼1 × 10^-4^ CFU per donor CFU). PCR amplification and sequence analysis of the junction site of the donor strain indicated that, on transfer, SGI-4 inserts into the same position on the chromosome (phe-tRNA locus) as the donor strain (data not shown). Furthermore, in the monophasic *S*. Typhimurium strain S04698-09 cultured in the presence of mitomycin C, PCR amplification and sequence analysis of an amplicon generated using specific outward facing primers at each end of SGI-4, was consistent with circularization of excised SGI-4 (data not shown).

### SGI-4 Is Characteristic of the Monophasic *S*. Typhimurium ST34 Clade

**FIGURE 3:**
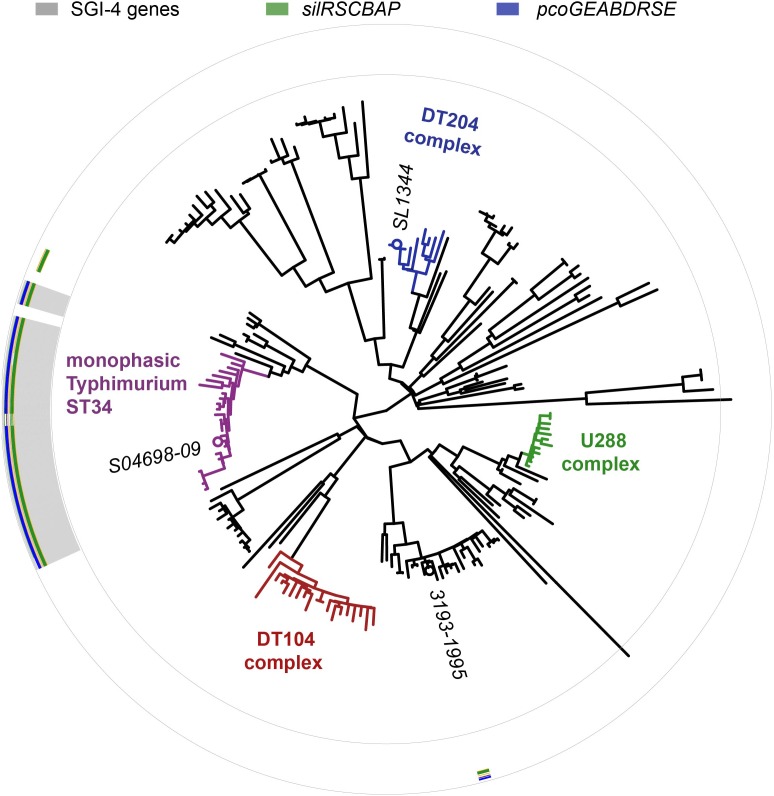
Distribution of SGI-4 in monophasic *S*. Typhimurium ST34 and representative strains of *S*. Typhimurium. A maximum likelihood tree constructed using sequence variation in the core genome of 24 monophasic *S*. Typhimurium ST34 strains and 153 representative strains of *S*. Typhimurium with reference to the whole genome sequence of strain SL1344 (accession FQ312003). Selected strains are identified (italicized text). The presence of sequences that mapped to 87 SGI-4 ORFs from S04698-09 are represented as filled boxes in 87 concentric circles: *sil* genes (green), *pco* genes (blue), or other SGI-4 ORFs (gray).

The SGI-4 sequence was present in 23 of 24 monophasic *S*. Typhimurium ST34 clade strains, but absent from all other strains, in a collection of representative *S*. Typhimurium (Figure 3). SGI-4 sequence was also present almost exclusively in isolates of the monophasic *S*. Typhimurium ST34 clade in the whole genome sequence of 1697 *S*. Typhimurium and monophasic variant *S*. Typhimurium isolates from human clinical cases in England and Wales during 2014 and 2015 (Supplementary Figure S1). Within the monophasic *S*. Typhimurium ST34 clade just 38 isolates (4%) lacked the SGI-4 sequence, and these were distributed sporadically throughout the clade in 28 small clusters or individual leaves of the phylogenetic tree (Supplementary Figure S1). In contrast, of 797 *S*. Typhimurium isolates that were present outside the monophasic *S*. Typhimurium ST34 clade, just five contained the SGI-4 sequence, and these all shared a recent common ancestor with the monophasic *S*. Typhimurium ST34 clade.

### SGI-4-Encoded *sil* Is Phylogenetically Distinct From the Chromosome-Encoded *cus* Genes of *Escherichia coli*

The nomenclature for copper RND-family efflux pump genes is confusing with *sil* and *cus* being used without reference to ancestry. A cluster of 18 ORFS on SGI-4 included 15 genes that exhibited sequence similarity to genes predicted to encode an RND-family efflux pump previously designated as either *cusRS cusCFBA* (Outten et al., 2001) or *silRSE silCBAP* (Gupta et al., 1999), and *pcoABDRSE pcoEG* (*pco* locus) involved in Cu and silver resistance. Alignment of the RND-family efflux pump genes of SGI-4 with sequences from previously characterized homologs indicated that the *cusRSCFBA* genes on the *E. coli* chromosome form a distinct outgroup from a closely related cluster that included the SGI-4 genes, that evolved from a common ancestor closely related to the *silRSE silCBAP* on pMG101 (Supplementary Figure S2). For this reason, we designated SGI-4 encoded RND-family efflux pump genes as *silRSE silCBAP* (*sil* locus).

### The *sil* Locus Is Rare in the Genus *Salmonella*

Investigation of the distribution of the SGI-4 *silA* genes in two sequence databases indicated that these genes are rare in the genus *Salmonella*, consistent with deletion early in the evolution of the genus, and infrequent reacquisition. Alignment using BLASTn of the *silA* gene on SGI-4 with 926 *Salmonella* genomes, one from each eBurst group (largely corresponding serovar) representing all known genotypic diversity of *Salmonella* (Alikhan et al., 2018), indicated that just 16 genomes (2%) contained an ortholog. In a second search of the ENA, the *silA* gene was in 3439 sequence entries, while the *invA* gene in 51026 data entries, indicating that *silA* was present in approximately 7% of *S*. Typhimurium genomes in the database.

### Enhanced Resistance of Monophasic *S*. Typhimurium ST34 to Cu *in vitro* Is Mediated by the *silCBA* Genes

**FIGURE 4:**
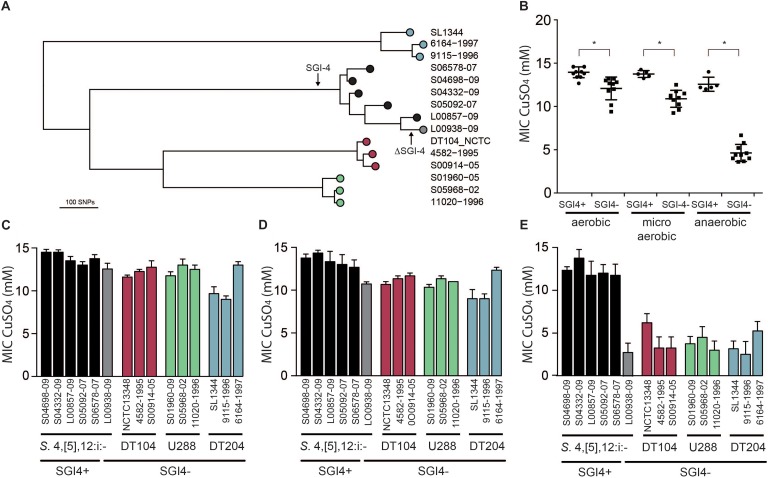
Phylogenetic relationship and Cu sulfate MIC of representative strains of monophasic *S*. Typhimurium ST34 and *S*. Typhimurium. **(A)** A mid-point rooted phylogenetic tree constructed using sequence variation in the core genome with reference to *S*. Typhimurium strain SL1344 whole genome sequence (accession FQ312003). Leaves of the tree corresponding to representative strains are labeled as filled circles color coded for SGI-4^+^ monophasic *S*. Typhimurium ST34 (black) and SGI-4^-^ monophasic *S*. Typhimurium ST34 (gray), and *S*. Typhimurium DT104 complex (red), U288 complex (green), and DT204 complex (blue) are shown. **(B)** Mean MIC for Cu sulfate of SGI-4^+^ (filled circles) and SGI-4^-^ (filled squares) monophasic *S*. Typhimurium ST34 and *S*. Typhimurium strains during aerobic, microaerobic and anaerobic culture. Bars indicate the mean MIC for Cu sulfate for each strain ± standard deviation in aerobic **(C)**, microaerobic **(D)**, and anaerobic **(E)** atmosphere. Bar colors match the tree leaf circles in **(A)**. ^∗^Indicates that groups indicated by lines were significantly different (*p* < 0.05).

In order to investigate the impact of the acquisition of SGI-4 on metal ion resistance, we compared the MICs of Cu sulfate for five strains of monophasic *S*. Typhimurium ST34 with three strains each of DT204, U288 and DT104 that lacked SGI-4, and a single monophasic *S*. Typhimurium ST34 strain that also lacked SGI-4 due to deletion (Figure 4). During culture in aerobic or microaerobic conditions the presence of SGI-4 had a small but significant impact on the MIC for Cu. However, under anaerobic conditions, the MICs for strains lacking SGI-4 decreased by around five-fold; in contrast, SGI-4-containing monophasic *S*. Typhimurium ST34 strains had similar MICs under both aerobic and microaerobic conditions.

**FIGURE 5:**
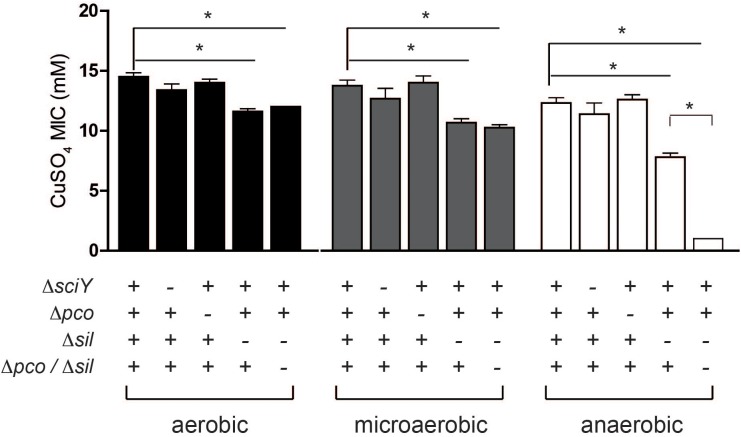
MIC for Cu sulfate of monophasic *S*. Typhimurium ST34 strain S04698-09 containing defined deletions in *sil* and *pco* genes. Bars indicate the mean MIC ± standard deviation of four biological replicates during culture in either aerobic, microaerobic or anaerobic conditions. The genotype with respect to *sciY* (negative control), *pco* and *sil* are indicated as present (+) or deleted and replaced with a *cat* gene (–). ^∗^ indicates the MIC was significantly different from wild type strain S04698-09 (*p* < 0.05).

SGI-4 has two clusters of genes predicted to encode an RND family efflux pump (*silCBA*), and a second that includes a multicopper oxidase system (*pcoABDE*), both previously implicated in resistance to Cu. To determine the relative role of these two loci in Cu resistance we determined the MICs of mutants of monophasic *S*. Typhimurium ST34 strain S04698-09 that had deletions of either *silCBA*, *pcoABC* or both these loci (Figure 5). When functional *silCBA* genes were present, deletion of *pcoABDE* genes alone had no effect on resistance to Cu under any of the conditions evaluated. Deletion of *silCBA* genes resulted in a small but significant (*p* < 0.05) decrease in the MIC for Cu under aerobic and microaerobic conditions, and a more substantial decrease under anaerobic conditions. However, even under anaerobic conditions, the effect of the *silCBA* deletion on the MIC for Cu was not comparable to the absence of SGI-4 in U288, DT104 and DT204 isolates which had a MIC that was below 5 mM Cu sulfate. Cu resistance at a similar level to these strains was only observed in monophasic *S*. Typhimurium ST34 strain S04698-09 when both *silCBA* and *pcoABDE* were deleted.

### The Presence of *silCBA* Genes on SGI-4 Alters Expression of *pcoA* and the Native Cu Homeostasis Gene *copA*

**FIGURE 6:**
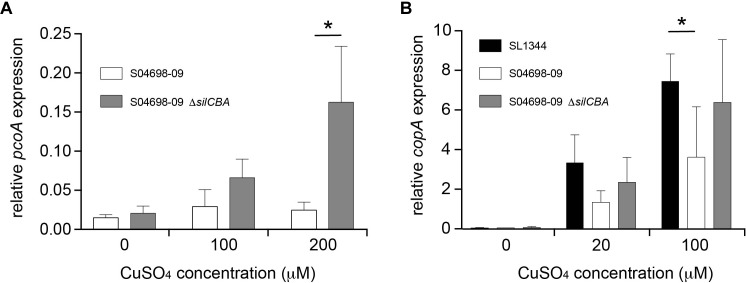
Presence of the *silCBA* genes decreases relative transcript abundance of *pcoA* and *copA*. The abundance of *pcoA* **(A)** or *copA* **(B)** transcript relative to the housekeeping gene *rpoD* quantified by quantitative RT-PCR from total RNA prepared from mid-log phase cultures in LB broth supplemented with Cu sulfate. Bars indicate mean relative transcript from five biological replicates ± standard deviation. ^∗^Indicates that groups indicated by lines were significantly different (*p* < 0.05).

To investigate any apparent redundancy of *pcoABDE* in the presence of *silCBA*, we determined the expression of *pcoA* in the presence or absence of *silCBA.* The expression of *pcoA* was not affected by increasing concentrations of Cu, unless the *silCBA* genes were deleted from monophasic *S*. Typhimurium ST34 strain S04698-09 (Figure 6A). In the absence of *silCBA* induction was around ten-fold that achieved in the absence of Cu in the growth medium.

To determine the impact of SGI-4 acquisition on typical Cu homeostasis in *S*. Typhimurium, we investigated the expression of the *copA* gene that encodes the native ATPase protein involved in transport of Cu from the cytoplasm into the periplasm in *Salmonella*. The expression of *copA* increased in a dose-dependent manner in the presence of Cu in *S*. Typhimurium strain SL1344, which lacked SGI-4, and in the monophasic *S*. Typhimurium ST34 strain S04698-09 that encoded the island. However, expression of *copA* was significantly higher in *S*. Typhimurium strain SL1344 than in monophasic *S*. Typhimurium ST34 strain S04698-09 during culture in media with 100 μM Cu. Deletion of *cusACFBA* genes in strain S04698-09 resulted in increased expression of *copA* approaching the expression level observed in SL1344 in 100 μM of CuSO_4_ (Figure 6B).

### Presence of the *silCBA* Genes Does Not Affect SodCI-Mediated Resistance to Macrophage Killing

We then tested the hypothesis that the presence of *silCBA* affected the function of SodCI, a periplasmic superoxide dismutase that uses Cu as co-factor that is supplied by the CopA ATPase Cu transporter and the periplasmic Cu binding protein CueP (Osman et al., 2013). SodCI dismutates oxygen free radicals produced by phagosome associated NADPH-dependent oxidase (Phox) of macrophages, contributing to intracellular survival of *Salmonella*. We expected that the expression of *silCBA* that encoded a copper RND efflux pump on SGI-4 would result in transport of Cu from the cytoplasm and periplasm to the external milieu (Gupta, 1994), depleting the pool of Cu available in the periplasm. Consistent with this, expression of *pcoA*, a periplasmic multicopper oxidase gene whose expression is controlled by PcoR that senses copper in the periplasm, was not induced during culture in 100 μM Cu, unless the *silCBA* was deleted from SGI-4.

**FIGURE 7:**
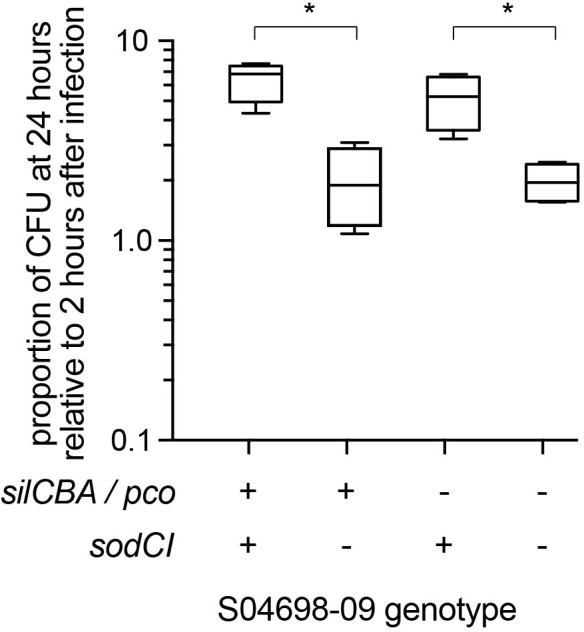
Net replication of monophasic *S*. Typhimurium ST34 in activated RAW macrophages is *sodCI* dependent but *sil* / *pco* independent. Box and whiskers plot indicating the mean number of bacterial CFUs 24 h post-infection, relative to 2 h post infection of wild type monophasic *S*. Typhimurium strain S04698-09 and otherwise isogenic strains containing deletions of the *sodCI* gene, the *sil*/*pco* genes together, or all three loci deleted. ^∗^Indicates that groups indicated by lines were significantly different (*p* < 0.05).

To test the hypothesis we infected gamma interferon-activated RAW macrophages with mutants of monophasic *S*. Typhimurium ST34 strain S04698-09 that encoded either *silCBA* and *sodCI*, or lacked one or both of these loci and determined the change in intracellular viable counts of *S*. Typhimurium in macrophages between 2 and 24 h post-inoculation (Figure 7). Strain S04698-09 exhibited a net replication of nearly ten-fold after 24 h in RAW macrophages, and deletion of the *sodCI* gene resulted in a small but significant (*p* < 0.05) reduction. This was consistent with previous reports that SodCI is required by the bacterium to evade the killing mechanisms of macrophages. However, deletion of the *silCBA* genes did not result in a significant decrease in net replication in RAW macrophages, but additional deletion of *sodCI* resulted in a similar decrease in net replication to that observed in the presence of the *silCBA* genes. These data are consistent with a fully functional SodCI gene in the presence of altered Cu homeostasis due to the presence of the *silCBA* genes.

## Discussion

For vertical transfer ICE undergo replication within the chromosome; for horizontal transfer they undergo excision from the genome (Johnson and Grossman, 2015). Horizontal transfer of an ICE involves common processes including excision from the host chromosome, circularization, conjugative transfer by a type IV secretion system (T4SS), and integration within a recipient genome at an attachment site (*attB*; Guglielmini et al., 2011). A number of ORFs exhibiting sequence similarity to genes encoding DNA processing enzymes that are predicted to be involved in excision, integration and conjugative transfer of DNA via a type IV secretion system (T4SSs) were also present on SGI-4. Together these data were consistent with the idea that SGI-4 is an ICE.

In an analysis of 1,124 taxonomically diverse complete prokaryotic genomes, 335 putative ICEs were detected based on the presence of T4SS and a relaxase gene in close proximity, indicating the widespread nature of these MGEs (Guglielmini et al., 2011). Despite the presence of multiple genes with sequence similarity to those with functions required for the transfer of ICEs, SGI-4 did not exhibit extensive similarity to previously characterized MGEs, and is currently not present in the ICEberg database (Liu et al., 2018). Seven complete genomes from diverse species of Enterobacteriaceae that were added to available databases since the analysis by Guglielmini et al. (2011), had between 75 and 99% nucleotide sequence identity with SGI-4 and between 41 and 76% shared core sets of genes with SGI-4. As is common for ICEs, core genes were interspersed with cargo genes with functions that were unrelated to mobilization but had the potential to modify the phenotype of the host bacterium (Wozniak and Waldor, 2010). In particular, genes involved in resistance to heavy metals were present on SLEs, with five containing at least one locus associated with resistance to Cu, silver, mercury or arsenic. Two SLEs from *Erwinia tracheiphila* and *Edwardsiella ictaluri* were particularly closely related to SGI-4, but contained unrelated cargo genes, highlighting the rapid divergence achieved by horizontal gene transfer in this family of ICE. Our analysis is consistent with SGI-4 evolving from a common ancestor of these ICEs by acquisition of *ars*, *sil* and *pco* cargo genes involved in resistance to metal ions.

With the exception of a small outbreak in a burns unit associated with a *S*. Typhimurium strain in the 1970s, historically, the *sil* and *pco* loci encoding resistance to silver and Cu, have rarely been associated with *Salmonella enterica* isolates. Indeed, we found that *sil* genes were present in just 2% of whole genome sequences representing the genotypic diversity of *S. enterica* and *S*. *bongori*, and 7% of more than 50,000 *Salmonella* whole genome sequences in the ENA. However, *sil* and *pco* genes, have been commonly associated with three lineages of monophasic *S*. Typhimurium in the past 20 years: the “European clone” (monophasic *S*. Typhimurium ST34); the “Spanish clone”; and the “Southern European clone” (Mourao et al., 2015; Mastrorilli et al., 2018). However, only 74 and 26% of the latter two, respectively, encoded the *sil* and/or the *pco* genes and these were plasmid-borne (Mourao et al., 2015), suggesting that they may be lost relatively frequently. In contrast, monophasic *S*. Typhimurium ST34 encodes *sil* and *pco* genes on the chromosome, and although on a MGE, we found these genes in 96% of the 797 clinical isolates from England and Wales that we evaluated. Furthermore, loss of SGI-4 and the *silCBA* and *pco* genes was sporadic, and mostly as singleton taxa on the phylogenetic tree, consistent with the absence of selection for their loss (Petrovska et al., 2016).

Given the general paucity of genes encoding Cu/silver RND efflux pumps in *Salmonella enterica* and the evolutionary history of the genus, the apparent lack of a selective advantage for the loss of *silCBA* genes may indicate an important role for these genes in the monophasic *S*. Typhimurium ST34 clone. Deletion of *cusCFBA* in the *Salmonella* ancestor is likely to have had a profound effect on Cu distribution in the cell because *Salmonella* has no alternative mechanism to remove Cu from cell. While *E. coli* can remove Cu from the cell entirely, *Salmonella* transports Cu from the cytoplasm, where it is especially toxic, to the periplasm, where it is detoxified by the Cu oxidase CueO or bound to the Cu binding protein, CueP, the major reservoir of Cu in *Salmonella* (Osman et al., 2010). CueP is thought to play an important role in supplying Cu to superoxide dismutase SodCI, important for survival in the face of the oxidative burst generated by macrophage. The evolution of *Salmonella* pathogenesis must, therefore, have proceeded in the context of fundamentally altered Cu homeostasis. The reintroduction of the *silCBA* locus on SGI-4 did indeed appear to alter the Cu distribution and levels in *Salmonella* as indicated by the lack of *pcoA* expression in the presence of the RND-family of efflux genes, which are regulated in response to Cu levels in the periplasm. The consequences of altered Cu levels in monophasic *S*. Typhimurium ST34 is not clear but, at least in RAW macrophages *in vitro*, the dependency of SodCI Cu as a cofactor did not affect resistance to macrophage killing. This is consistent with the report that limitation of Cu in the periplasm by deletion of the *copA* and *golT* genes, and the supply of Cu to SodCI by deletion of the *cueP* gene in S. Typhimurium had no effect on systemic infection in the murine model of infection (Fenlon and Slauch, 2017).

Strong selection pressure may be important for retention of SGI-4 since our data is consistent with its presence altering Cu distribution and levels. Monophasic *S*. Typhimurium ST34 is primarily associated with pigs, and it has been suggested that the success of this clone may, in part, have been driven by the extensive use of Cu as a growth promotor in pig rearing (Mourao et al., 2015; Petrovska et al., 2016). Consistent with this idea, the presence of SGI-4 is correlated with enhanced resistance to Cu; this appears to be particularly apparent under anaerobic conditions which are similar to those encountered by *Salmonella* in the host intestinal tract. *Salmonella* strains that lacked SGI-4, exhibited MICs for Cu of around 2–3 mM under anaerobic conditions, which is approximately 15% of the values achieved under aerobic conditions. Importantly, this is in the range of the concentrations of Cu found in pig manure effluent and sludge on farms where Cu-supplemention of diet was common (Nicholson et al., 1999; Bolan et al., 2003; Cang et al., 2004). The presence of SGI-4, however, increased the MIC for Cu (under anaerobic conditions) by approximately 500%, elevating it to levels above the concentrations likely to be encountered on pig farms (Nicholson et al., 1999; Bolan et al., 2003; Cang et al., 2004). The *sil* genes encoded on SGI-4 were entirely responsible for the observed increase in MICs for Cu, since deletion of *pco* genes alone did not result in a decrease in the MICs. The *pco* locus encodes a multiCu oxidase which is thought to detoxify the more damaging Cu^+^ to Cu^2+^ ions by oxidation (Lee et al., 2002). Monophasic *S*. Typhimurium ST34 encodes a native Cu oxidase, CueO, and the presence of this protein may, in part, mask the activity of the *pco* locus. However, the *pco* genes were capable of enhancing resistance to Cu in the absence of *silCFBA*. Furthermore, since there was no evidence for loss of *pco* genes during clonal expansion of the monophasic *S*. Typhimurium ST34 clade, it remains possible that the Pco system may play a more prominent role in Cu resistance under environmental conditions that we did not evaluate in our *in vitro* experiments.

## Author Contributions

RK, PB, and OC conceived the study. PB and OC performed the experiments and generated the data. TD and LP provided materials and expert advice. RK, PB, and OC drafted the manuscript. All authors analyzed the data, provided critical input into the final manuscript, and approved the final version.

## Conflict of Interest Statement

The authors declare that the research was conducted in the absence of any commercial or financial relationships that could be construed as a potential conflict of interest.

## Footnotes

1 https://github.com/tseemann/snippy

## Supplementary Material

Figure S1 Distribution of SGI-4 in monophasic *S.* Typhimurium ST34 and representative strains of *S*. Typhimurium. A maximum likelihood tree constructed using sequence variation in the core genome of 1814 monophasic *S.* Typhimurium ST34 strains or *S.* Typhimurium isolates with reference to the whole genome sequence of strain SL1344 (accession FQ312003). The presence of sequence in each genome that mapped to 787 SGI-4 ORFs from S04698-09 are represented as filled boxes in 87 concentric circles: *sil* genes (green), *pco* genes (blue), or other SGI-4 ORFs (gray).

Figure S2 A maximum likelihood tree showing the relationship of *cus* and *sil* loci.

Table S1 Oligonucleotide primers used for the construction of mutant strains of *Salmonella*, determination of genotype, or qRT-PCR.

Table S2 Isolate name, accession number and the figure(s) in which the data were used to construct maximum likelihood trees.

Table S3 Gene model for SGI-4 including gene designation, annotation, SLE core genome and nucleotide sequence of predicted coding sequences.
