# Supplementary figures and images for: SGI-4 in Monophasic Salmonella Typhimurium ST34 Is a Novel ICE That Enhances Resistance to Copper
Source: Front Microbiol. 2019 May 24;10:1118. doi: 10.3389/fmicb.2019.01118 (PMC6543542; doi:10.3389/fmicb.2019.01118)

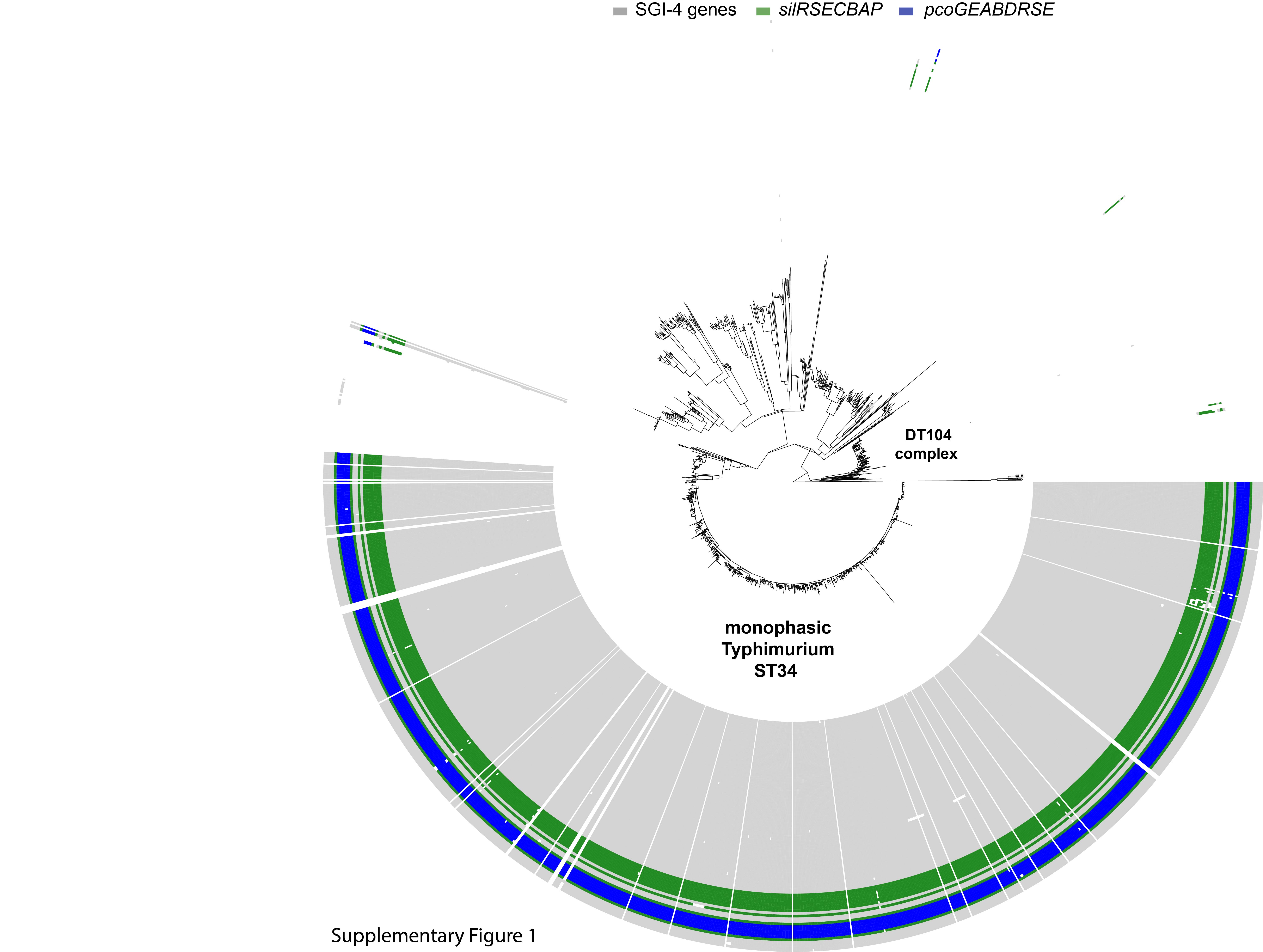

Supplement: Figure S1 — Distribution of SGI-4 in monophasic S. Typhimurium ST34 and representative strains of S. Typhimurium. A maximum likelihood tree constructed using sequence variation in the core genome of 1814 monophasic S. Typhimurium ST34 strains or S. Typhimurium isolates with reference to the whole genome sequence of strain SL1344 (accession FQ312003). The presence of sequence in each genome that mapped to 787 SGI-4 ORFs from S04698-09 are represented as filled boxes in 87 concentric circles: sil genes (green), pco genes (blue), or other SGI-4 ORFs (gray). [file Image_1.JPEG]

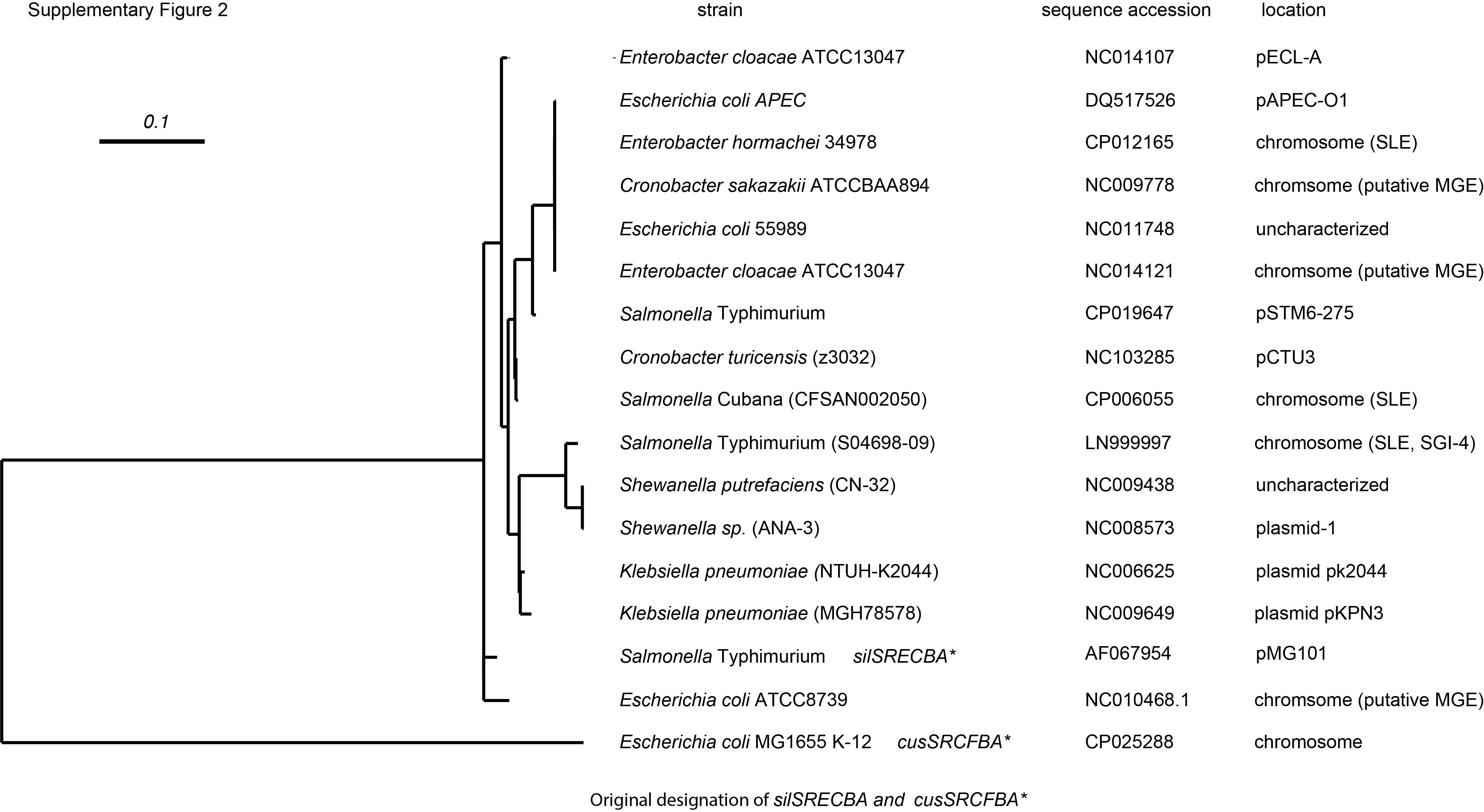

Supplement: Figure S2 — A maximum likelihood tree showing the relationship of cus and sil loci. [file Image_2.JPEG]
